# Perspectives on the Evolution of Porcine Parvovirus

**DOI:** 10.3390/v9080196

**Published:** 2017-07-26

**Authors:** Woo-Taek Oh, Ri-Yeon Kim, Van-Giap Nguyen, Hee-Chun Chung, Bong-Kyun Park

**Affiliations:** 1 Department of Veterinary Medicine Virology Lab, College of Veterinary Medicine and Research Institute for Veterinary Science, Seoul National University, Seoul 08826, Korea; mike0202@snu.ac.kr; 2 Queen’s University, Pathology and Molecular Medicine, Kingston, ON 613-343, Canada; laurary.kim@gmail.com; 3 Department of Veterinary Microbiology and Infectious Diseases, Faculty of Veterinary Medicine, Vietnam National University of Agriculture, Hanoi 100000, Vietnam; nguyengiap83@gmail.com

**Keywords:** porcine parvovirus, South Korea, phylogenetic analysis, evolution

## Abstract

Porcine parvovirus (PPV) is one of the main causes of porcine reproductive failure. It is important for swine industries to understand the recent trends in PPV evolution. Previous data show that PPV has two genetic lineages originating in Germany. In this study, two more genetic lineages were defined, one of which was distinctly Asian. Additionally, amino acid substitutions in European strains and Asian strains showed distinct differences in several regions of the *VP2* gene. The *VP1* gene of the recent PPV isolate (T142_South Korea) was identical to that of Kresse strain isolated in the USA in 1985, indicating that modern PPV strains now resemble the original strains (Kresse and NADL-2). In this study, we compared strains isolated in the 20th century to recent isolates and confirmed the trend that modern strains are becoming more similar to previous strains.

## 1. Introduction

Porcine parvovirus (PPV) was first isolated in Germany in 1965 as a contaminant of porcine primary cell culture that was used for the propagation of classical swine fever virus [1]. Porcine parvovirus is a small, non-enveloped virus with a single stranded DNA genome structure containing approximately 4–6.3 kb [2]. PPV has two open reading frames (ORFs) that comprise the non-structural protein (NS1), viral protein 1 (*VP*1), and major structural protein (*VP*2). The *VP*2 protein is the main target of neutralizing antibodies against PPV [3]. PPV is a member of family *Parvoviridae*, which includes two subfamilies, *Parvovirinae*, which infects vertebrates, and *Desovirinae,* which infects arthropods [4]. Furthermore, the subfamily *Parvovirinae* can be divided into eight genera: *Amdoparvovirus*, *Aveparvovirus*, *Dependoparvovirus*, *Erythroparovirus*, *Protoparvovirus*, *Bocaparvovirus*, *Copiparvovirus*, and *Tetraparvovirus* [5]. Porcine Parvovirus 1 (PPV1), which belongs to the genus *Protoparvovirus*, is a well-known infectious agent that causes reproductive failure in swine herds [6], and the clinical symptoms include fetal death, mummification, and the reoccurrence of estrus [7]. PPV outbreaks have occurred in many countries, in which swine industries have suffered serious economic losses [8]. Although recombinant and modified live-virus vaccines are available, several cases of PPV have been reported in various countries [9,10]. PPV was prevalent in South Korea and was continuously surveyed due to significant losses in the swine industry. Although the vaccine reduced infection rates dramatically [11], there have been no recent investigations of PPV. The nucleotide substitution rate of PPV was approximately 10^−5^ substitutions per site per year for the *NS1* gene and 10^−4^ substitutions per site per year for the *VP1* and *VP2* genes. These rates are similar to the nucleotide substitution rates in RNA viruses [3]. Consequently, a need for updated PPV vaccines has been suggested in several studies [1]. Therefore, we hypothesized that variations in PPV could be found in South Korea, and that PPV could re-emerge due to the rapid evolution of the virus. In the present study, we investigated the prevalence of PPV in South Korea by analyzing nucleic acids isolated from lung tissue samples collected from 2013 to 2016. These samples had caused abortions in pigs and were sent for identification. Furthermore, we characterized the genome of the PPV positive sample by genomic sequencing. Based on its sequence, we analyzed the phylogenetic study of the isolate and compared it to strains from South Korea and other countries.

## 2. Materials and Methods

### 2.1. Sample Collection, Extraction of Viral DNA, Detection, Sequencing, and Isolation of Porcine Parvovirus

Seven hundred and one internal tissue samples were collected from five different age groups (from fetuses to adult pigs) located in South Korea from March 2013 to December 2016. Samples were sent to Seoul National University School of Veterinary Medicine Virology Lab for diagnosis of the infectious agents that caused abortions in domestic pigs from nine different provinces in South Korea. DNA extraction was performed using an RNA/DNA Extraction kit (Invitrogen, Carlsbad, CA, USA) according to the manufacturer’s instructions and the extracted samples were stored at −20 °C. To detect PPV, we designed primers (PPV P1 and PPV P6) targeting 250 bp of ORF2 based on the alignments of 42 genomes of PPV found in the GenBank. The thermal profile included initial denaturation at 94 °C for 5 min, followed by 40 cycles at 94 °C for 30 s, 55 °C for 30 s, 72 °C for 30 s, and a final extension at 72 °C for 5 min. All PCR (Polymerase Chain Reaction) products were separated by electrophoresis on a 2% agarose gel and target bands were excised and purified using the QIAquick gel extraction kit (Qiagen Inc., Germantown, MD, USA). Positive samples were double checked using real-time PCR. Real-time PCR was carried out in 96-well plates and standards were run in triplicate. Each reaction consisted of a total volume of 25 μL, including 12.5 μL of the SYBR Green real-time PCR master mix (Applied Biosystems, Foster City, CA, USA), including 7.5 μL of distilled water, 1 μL of each primer, and 2 μL of the sample or standard DNA. Amplification and quantification reactions were performed using the ABI 7500 Fast Real Time PCR system (Applied Biosystems) under the following conditions: 2 min at 50 °C, 10 min at 95 °C, 40 cycles of 12 s at 95 °C, and 1 min at 60 °C [12]. Samples were considered negative if no threshold cycle was detected in 40 amplification cycles. To isolate PPV, PK15 cells were grown in Dulbecco’s Modified Eagle’s Medium (DMEM) supplemented with 10% fetal bovine serum and antibiotics (penicillin 100 IU/mL). To adapt the virus, 0.1% trypsin was used up to passage 10. For the complete genome sequencing of PPV, six pairs and one single primer were designed based on the alignments of 23 complete PPV genomes from GenBank. All primers used in this study are listed in Table 1. 

Specific PCR bands were purified using QIAquick Gel Extraction Kit (Qiagen Inc.), cloned using the TA cloning kit (Topcloner TA kit; Enzynomics, Daejeon, Korea) and subsequently transformed into competent *Escherichia coli* cells (DH5α). The purified recombinant plasmids were sequenced by Macrogen Inc. (Seoul, Korea). The T142_South Korea strain sequenced in this study can be found in GenBank accession no. KY994646.

### 2.2. Phylogenic Analysis and Evolutionary Rate Estimation

**Figure 1:**
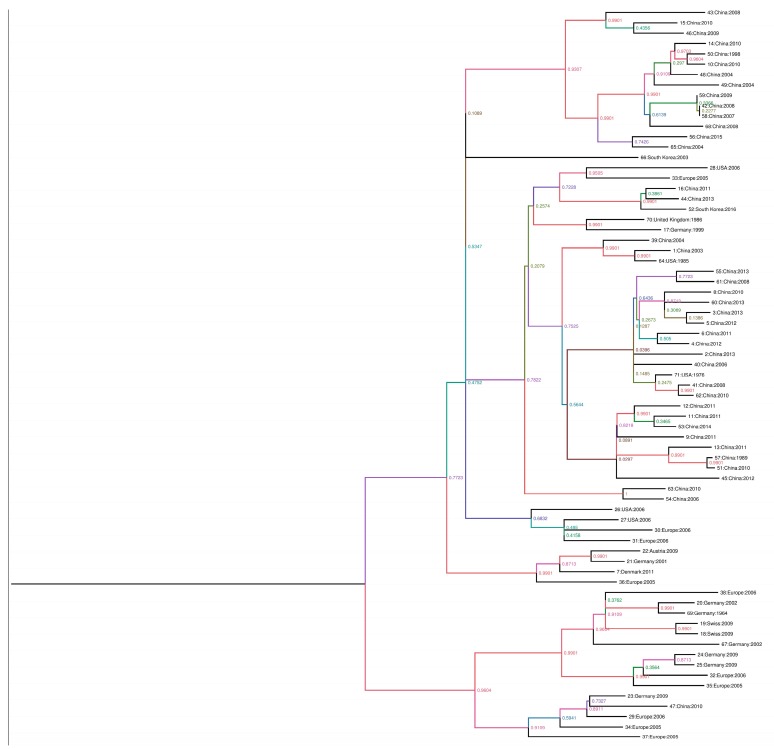

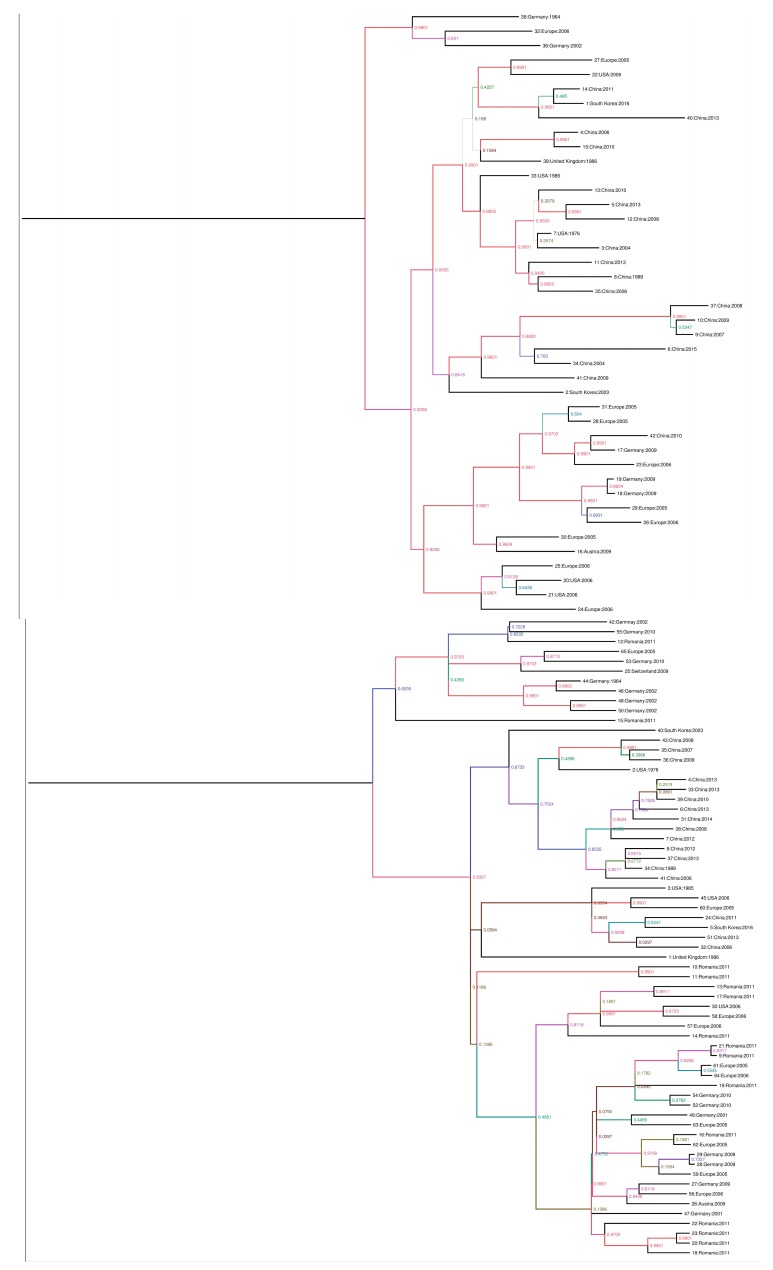

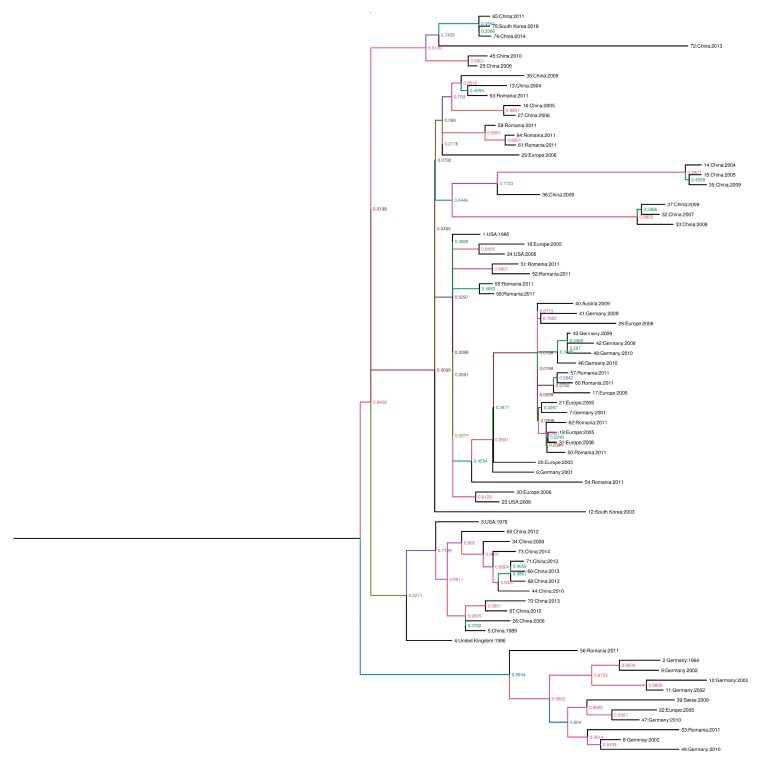
Phylogenic trees created with the non-structure protein gene (*NS1*), viral protein gene (*VP1*), and major structural protein (*VP*2) sequences. The scale axis indicates the distance in years and posterior probabilities are indicated according to the color of the branches. The phylogeny was estimated using the Bayesian continuous-time Markov chain (CTMC) method with Beast version 1.8.2. The phylogenic tree was visualized and edited using FigTree version 1.4.2, in the following order: 1: *NS1*, 2: *NS1*, *VP*1, *VP*2, 3: *VP1*, 4: *VP*2.

For the phylogenic analysis, the complete sequences were downloaded from GenBank and aligned using the ClustalW program in the BioEdit software version 7.0.9 [13]. Phylogenic trees were inferred by the maximum-clade credibility method (nucleotide sequences) implemented in Beast version 1.8.2. The branches of the maximum clade credibility tree were colored according to the most probable location state of their descendent nodes. To estimate the substitution rates per site per year and the time in the *NS1*, *VP*1 and *VP2* genes of PPV, we aligned 71 complete NS1, 65 complete *VP*1, and 75 *VP*2 sequences from GenBank and aligned by using the ClustalW program in the software BioEdit software version 7.0.9 [13]. Root-to-tip analysis was conducted using TemPest version 1.5 to assess whether there was sufficient temporal signal to proceed with the phylogenic molecular clock analysis. Sequences that were not suitable for analysis were excluded in the molecular clock analysis [14]. Rates of nucleotide substitutions per site per year and time to most recent common ancestor (TMRCA) were estimated using the Bayesian framework [15], which was applied to reconstruct the spatial-temporal diffusion history of PPV. In brief, the spatial diffusion of the time-scaled genealogy is modeled as a standard continuous-time Markov chain (CTMC) process over discrete sampling locations. A Bayesian stochastic search variable selection (BSSVS) approach, which allows the exchange rates in the CTMC to be zero with some prior probability, was used to find a parsimonious set of rates explaining the diffusions in the phylogeny. The analysis was performed using Beast package v1.8.2 under the following assumptions (i) a codon based SRD06 nucleotide substitution model, (ii) a constant population size for the coalescent prior, and (iii) the molecular clock model of uncorrelated lognormal distribution. The analysis was run for 100 million chains, sampling every 10,000 generations. The phylogenic trees were summarized with TreeAnnotator and were depicted using FigTree [16]. Groupings with posterior probabilities over 0.90 were considered to be clusters and those with posterior probabilities less than 0.90 were considered to be clades. This process was also performed using 75 sequences of the complete *VP2* gene and 71 sequences of the complete *NS1* gene (Figure 1).

### 2.3. Molecular Structure of the T142_South Korea Strain

For the estimation of amino acid substitutions, a 3-D model of PPV *VP*2 was drawn using the cartoon technique [17]. The sites indicated specific amino acids substitutions in the T142_South Korea, Kresse, NADL-2, Challenge, Vaccine IDT, and South Korea 2003 strains. The coordinates were retrieved from the National Center for Biotechnology Information (NCBI) Structure database accession number: 1K3V [17].

### 2.4. Recombination Analysis and Estimates of Amino Acid Mutation

For the detection of potential recombination events, we aligned the complete *NS*1, *VP*1, and *VP*2 sequences of 42 PPV strains and used a recombination detection program (RDP version 4.460). X-over automated RDP analysis was used to identify recombination points within the PPV genome. For estimates of amino acid mutations, we compared the similarity of 75 *VP*2 sequences with the T142_South Korea strain using the DNAstar (Lasergene, Madison, WI, USA) program and aligned each of sequences using the ClustalW program in BioEdit version 7.0.9 [13].

## 3. Results and Discussion

### 3.1. Nucleic Acid Detection of Porcine Parvovirus and Phylogenetic Analysis

**Figure 2:**
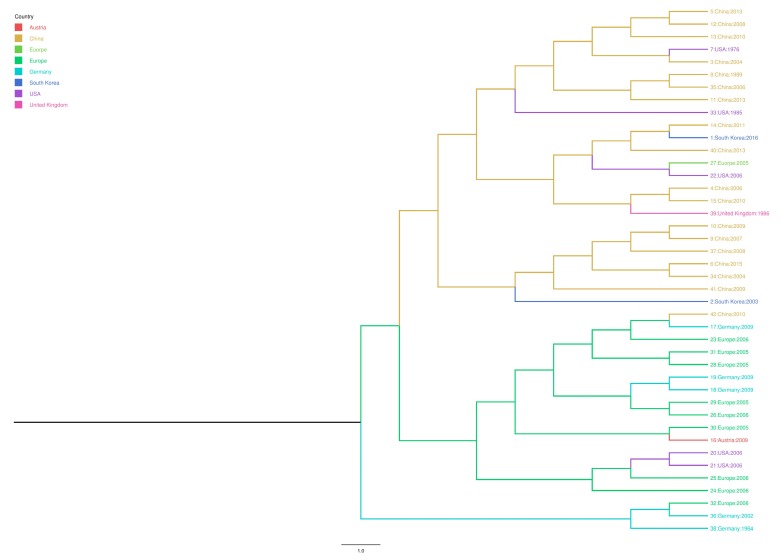
Bayesian time-scaled phylogeny of porcine parvovirus (PPV) (*NS1*, *VP1*, and *VP2* genes) with inferred geographical location states. The branches of the maximum clade credibility tree were colored according to the most probable location state of their descendent nodes. The color codes are defined in the insert legend.

Only one sample collected from 2013 to 2016 was positive for PPV. This sample was from a lung of a sow from a farm in Gyeonggi Province of South Korea. The complete sequence of T142_South Korea (accession number: KY994646) was 4762bp. A maximum clade credibility tree of the complete *NS1*, *VP1*, and *VP2* sequences was constructed. Our results showed that strain T142 South Korea was closest to the China 2011 (accession number: JN860197.1) and China 2013 strains (accession number: KF742500.2). Additionally, the 2003 South Korea strain (accession number: AY390557.1) formed a distinct root that was slightly different than the roots of the Chinese strains (Figure 2).

**Figure 3:**
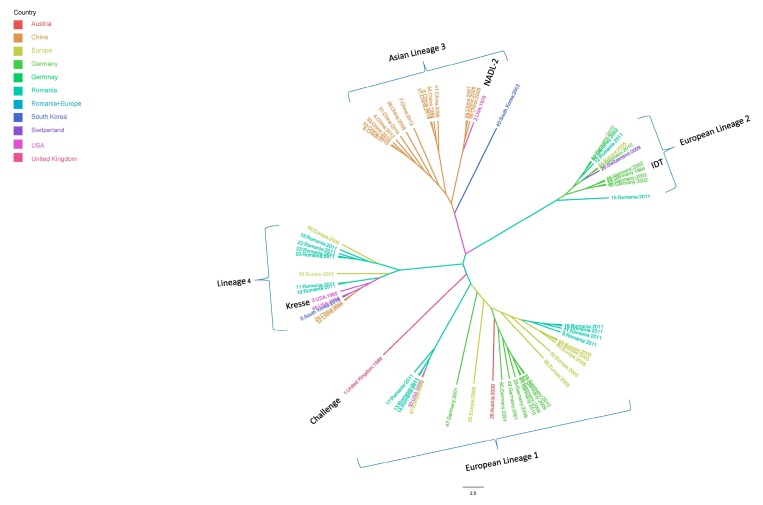
Maximum clade credibility tree for the complete *VP1* gene of PPV using spatial diffusion of the time-scaled genealogy modeled as a standard continuous-time Markov chain (CTMC) process over discrete sampling locations, using Beast Version 1.8.2. Scaled phylogeny of PPV inferred the geographical location states. The branches of the maximum clade credibility tree are colored according to the most probable location state of their descendent nodes. The color codes are defined in the insert legend. The phylogenetic tree was visualized and edited using FigTree version 1.4.2.

The maximum clade credibility tree constructed using the *VP1* and *VP2* sequences showed four major distinct lineages. Group 1 consisted of European strains that were similar to the Challenge strain (accession number: AY684866.1) isolated from the United Kingdom in 1986. Group 2 consisted of other European strains that were similar to the German vaccine strain IDT (accession number: AY684872.1). Thus, PPV evolution in Europe resulted in approximately two lineages. Group 3 consisted of Asian strains mostly from China and that were similar to the vaccine strain NADL-2 (accession number: NC_001718.1). Group 4 had no regional specificity and consisted of strains from various countries in Europe, the USA, and Asia. These strains were similar to the Kresse strain (accession number: U44978.1), which was isolated in the USA in 1985 (Figure 3).

Since the Asian lineage departed completely from the European lineage, we can assume that there were clear differences between sequences of European strains and Asian strains. Consequently, we compared amino acid substitutions between the European and Asian strains and determined the locations of each mutation in the *VP2* gene (Table 2).

As a result, many regions, including the sequences required for replication efficiency in tissue culture (aa 378, 383, 436 and 565) [5], showed different evolutionary patterns between the European and Asian strains.

### 3.2. Isolation and Characterization of Strain T142_South Korea

**Figure 4:**
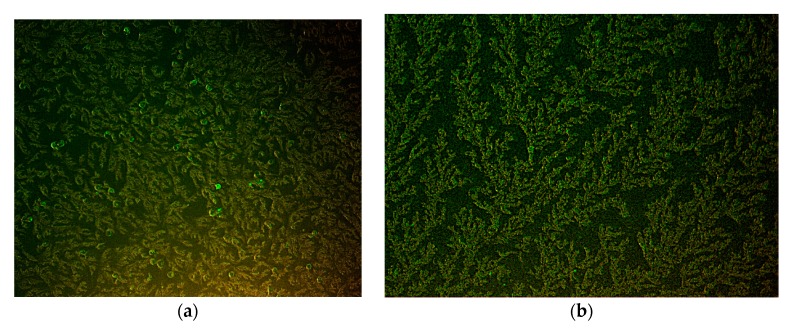
Indirect immunofluorescence of assay of PK-15 cells infected with PPV. (**a**) Infected cells fluoresced green, and (**b**) control cells stained in the same manner (200× magnification).

The virus was isolated successfully in the PK15 cell line. The cytopathic effects on the cells were observed (from passage 3) and an indirect immunofluorescence assay was performed to identify PK15 cells (from passage 10) infected by PPV. Positive nuclear fluorescence five days post infection was used as confirmation (Figure 4).

### 3.3. Recombination and Structural Analysis of Porcine Parvovirus Strain, T142

**Figure 5:**
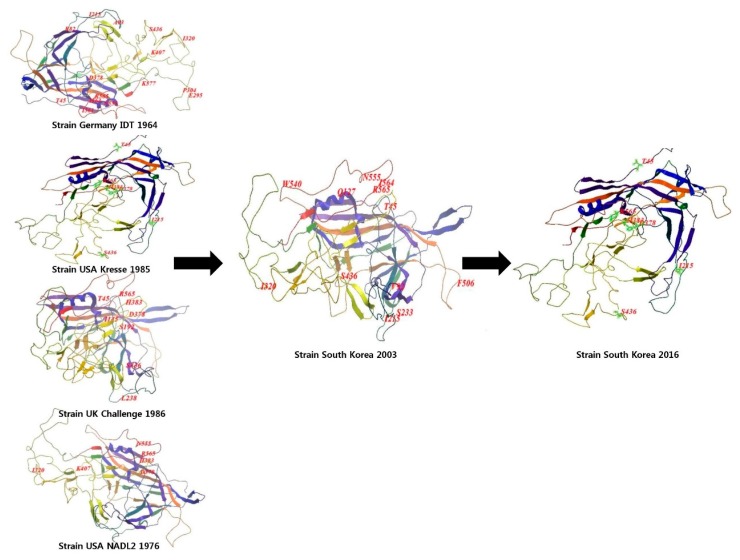
Cartoon structure of NADL-2, Kresse, Challenge, Germany Vaccine Tornau, South Korea 2003 (accession number: AY390557.1), and T142_South Korea strains drawn chronologically with Mol soft Mol Browser 3.8–5 according to the original publication from the National Center for Biotechnology Information (NCBI) Structure database [17]; accession number: 1K3V.

To determine whether recombination occurred in the older PPV strains, 42 complete *NS1, VP1,* and *VP2* sequences were collected, and recombination analysis was performed using the RDP program. We did not find any recombination sites in T142_South Korea. Additionally, to observe the *VP2* amino acid mutations clearly, we visualized the structure of the *VP2* protein using the cartoon technique [17]. The region where mutations occurred in the following strains are shown in Figure 5: 2003 South Korea (accession number: AY390557.1), T142_South Korea, NADL-2, Kresse, Challenge, and IDT vaccine, based on 1K3V. Strains NADL-2, Kresse, Challenge, and IDT vaccine strains were selected for the comparison because they are representative strains isolated in 20th century used for many studies of PPV in the past and vaccines manufactured afterward have followed them.

Interestingly, the 2003 South Korean strain was completely distinct from the NADL-2, Kresse, Challenge, and German Vaccine IDT strains. However, the *VP2* region of T142 was identical to that of Kresse. Thus, we inferred that the nucleotide substitutions in PPV accumulated to a certain level before reverting back to the original strains and the evolutionary patterns briefly depicted in Figure 5 were the actual patterns seen mostly in recently isolated PPV strains.

### 3.4. Evolution Rates in Recent Porcine Pravoviruses Including T142 Strain.

The CTMC method [13] was used to estimate the rate of evolution. The mean rate was estimated to be 9.71 × 10^−6^ substitutions/site/year for the *NS1* gene, 3.27 × 10^−5^ for the *VP1* gene, 5.47 × 10^−5^ for the *VP2* gene, and 4.25 × 10^−5^ for the complete sequence (Table 3).

These substitution rates are fast compared to those of other DNA viruses, but slightly lower than substitution rates found in previous study [9]. Thus, we inferred that the substitution rate decreased considering recent studies of PPV in China found that the amino acid substitution rate in Chinese strains was lower than that in European strains. The mean evolutionary rate for Chinese strains was 1.139 × 10^−5^, which is approximately 10 times less than the nucleotide substitution rate in European strains.

### 3.5. Homology Comparison between Porcine Parvovirus Strains before and after the 21th Century

Similarity tables for the amino acid mutations in the *VP1* and *VP2* genes were constructed using the DNAstar (Lasergene, Madison, WI, USA) program. In *VP2*, amino acid mutations ranged from 0 to 5.6%, and nucleotide mutations ranged from 0 to 2.3%. In *VP1*, amino acid mutations ranged from 0 to 4.0%, and nucleotide mutations ranged from 0 to 2.0%. The average similarity values between strains isolated before the 21st century and those isolated afterward were compared and classified into four groups for five years. The mutations were determined in 28 highly variable regions of the *VP2* gene, and are shown in Table 4 and Table 5 and the detailed information about the amino acid substitutions made between strain Kresse, NADL-2, and 74 other strains were given in Supplementary Tables S4-1 and S4-2.

We found that, compared to the NADL-2 and Kresse strains, the average nucleotide substitutions (site numbers per strain) decreased continuously after 2005, indicating that the nucleotide substitutions were becoming more similar to those of past strains such as NADL-2 and Kresse. In addition, the eight most variable locations compared to Kresse were aminoacid (aa) 215, 228, 233, 320, 383, 414, 419, and 436. Compared to NADL-2, they were aa 45, 555, 436, 407, 215, 320, 419, and 414. Additionally, we compared similarity values between strains isolated before the 21st century and strains isolated afterward and then divided them into four groups within five years. The average similarity value increased from 98.9853% to 99.1969% in nucleotide sequences and 98.0223% to 98.5731% in amino acid sequences (Table 6) and the detailed data of similarity values between nucleotides and amino acids of each strain were given in Supplementary Table S3.

These results indicate that the present strains are becoming more similar to earlier strains. We concluded that nucleotide substitutions are still occurring in most strains and that they are reverting back to original strains isolated more than 30 years ago rather than becoming variants to the vaccine strain (NADL-2). If the vaccine is efficient, viruses similar to the vaccine strain will be nearly eradicated and genetic diversity will decrease due to natural selection and the adaptation of the viruses. Therefore, we anticipate that PPV will diverge evolutionarily from the vaccine strain. However, PPV nucleotide substitutions have not followed natural selection and instead have defied usual evolutionary patterns.

## 4. Conclusions

In conclusion, our study provides insight into the evolution of PPV. The Asian lineage was completely distinct from the European lineage and formed its own evolutionary clade. It is remarkable that amino acid mutations in PPV are becoming more similar to those of the vaccine strain (NADL-2). The *VP1* and *VP2* genes, as well as specific amino acid substitutions, are known to play an important role in virulence and are responsible for host tropism in tissue culture cells. The evolutionary rate of PPV and the necessity for vaccines are closely related to the *VP1* and *VP2* genes. Therefore, PPV strains that show re-emerging patterns similar to past strains are crucial for the future of the domestic pig industry. Similar re-emerging patterns to past strains can easily lead to the idea that need for the updated PPV vaccines is not essential but nucleotide shift is not the only factor affecting prevention of the disease. Additionally, the four genetic lineages determined by the *VP1* and *VP2* genes implies that PPV evolution may be closely related to regional and environmental factors, so it may be essential for us to keep examining the evolutionary patterns of PPV for the prevention of the disease.

## Figures and Tables

**Table 1:** Details of primers used in this study.

Primer Name	Sequence	Product Size (Binding Position to Accession Number KY994646)
PPV START F	GGCTGAAAAGAGGCGGGAAATT	503 bp (1–503)
PPV R	CAGTAGCAGTCACTTGGACTTAG	
PPV NS1 F1	ATGGCAGCGGGAAACACTTACT	1005 bp (160–1165)
PPV NS1 R1	TGTTCTTGCTAGAGTAAGAGTTG	
PPV NS1 F2	ACCGGAGGAGAAAATTTAATCA	1020 bp (1100–2120)
PPV NS1 R2	TGCACAGTTTTCACCAAAGCAGG	
PPV VP1 F1	TTGGTCGGAAATAGAAACCGACATA	1040 bp (2070–3110)
PPV VP1 R1	TTGTTCAAAACTAACTAAGTTT	
PPV VP1 F2	GCAGTTAATATCCAACAACATG	1020 bp (3050–4070)
PPV VP1 R2	CCCATATTTGACCATTTGGAAATA	
PPV VP1 F3	ACCATTAACAGCACTAAACAATAC	400 bp (4020–4420)
PPV VP1 R3	CTAGTATAATTTTCTTGGTATAA	
PPV END	CTAAAGACATAAGGTCATATAAGT	742 bp (4020–4762)
PPV RT F	AGGTAAGAAGATCGCCGAGAAA	110 bp (2550–2660)
PPV RT R	AGATGTCCCTTTAGCTTTTTTTTTAGC	
PPV P1	ATACAATTCTATTTCATGGG	330 bp (1330–1660)
PPV P6	TATGTTCTGGTCTTTCCTCG	

**Table 2:** Distinct amino acid regions in *VP*2 in European and Asian strains.

Amino Acid Location	Europe Group (*n* = 42)	Asia Group (*n* = 29)
20	A (10) T (32)	T (29)
82	K (10) R (32)	R (29)
144	E (42)	A (6) E (23)
215	I (1) T (41)	I (15) V (3) T (11)
228	E (20) Q (22)	Q (29)
304	T (10) P (32)	P (29)
378	G (42)	D (9) G(20)
383	H (1) Q (41)	H (16) Q (13)
414	S (18) A(24)	S (1) A(28)
419	Q (20) E(22)	E (29)
436	T (23) A (4) P(15)	S (12) A(9) H(1) P(7)
555	N (40) K (2)	N (21) K (8)
565	K (41) E (1)	K (20) R (9)

**Table 3:** Information on the evolutionary rate measured in this study and its analyzing method.

Dataset	Number of Sequence Used	Clock Model	Mean Rate	95% HPD Interval
NS1 complete	71	UCLD	9.71 × 10^−6^	2.30 × 10^−5^
				8.72 × 10^−9^
VP1 complete	65	UCLD	3.27 × 10^−5^	7.06 × 10^−5^
				5.83 × 10^−7^
VP2 complete	75	UCLD	5.47 × 10^−^^5^	1.05 × 10^−4^
				1.49 × 10^−5^
NS1, VP1,VP2 complete	42	UCLD	4.25 × 10^−5^	8.01 × 10^−5^
				7.51 × 10^−6^

UCLD: uncorrelated relaxed clock model with an underlying lognormal distribution.

**Table 4:** Nucleotide substitutions occurred in 28 highly variable regions in the *VP2* gene of 74 strains compared to strain Kresse (accession number: U44978.1). The average total variant site number/strain was estimated according to four groups classified into five years. The variance in every region was measured, and the eight most variable regions were checked.

Average Total Variant Site Numbers/Strain														
			~2000 (*n* = 4)			2001~2005 (*n* = 17)			2006~2010 (*n* = 27)			2011~2016 (*n* = 26)		
Variant Number/Strain			5.0/strain			5.88/strain			4.70/strain			3.28/strain		
**Number of Strains Variance Occurred by Region**														
Location	20	45	82	93	101	144	215	226	228	233	238	304	320	366
Number	10	8	10	4	3	6	19	3	20	17	4	10	21	7
Location	378	383	391	407	414	419	436	439	521	550	555	564	565	570
Number	9	17	9	16	19	20	54	3	5	4	10	7	10	7

**Table 5:** Nucleotide substitutions occurred in 28 highly variant regions of the *VP2* gene of 74 strains compared to strain NADL-2 (accession number: NC_001718.1). The average total variant site number/strain was estimated according to four groups classified into five years. The variance in every region was measured and the eight most variable regions were checked.

Average Total Variant Site Numbers/Strain															
			~2000 (*n* = 4)			2001~2005 (*n* = 17)			2006~2010 (*n* = 27)			2011~2016 (*n* = 26)			
Variant Number/Strain			6.5/strain			9.529/strain			7.9629/strain			6.4615/strain			
**Number of Strains Variance Occurred by Region**															
Location	20	45	82	93	101	144	215	226	228	233	238	304	320		
Number	9	66	9	4	3	6	59	3	10	17	4	11	56		
Location	366	378	383	391	407	414	419	436	439	521	550	555	564	565	570
Number	7	9	17	9	61	18	20	61	3	5	4	63	7	10	7

**Table 6:** Comparison of *VP2* genes (nucleotide and amino acids) of five strains isolated in the 20th century and 70 strains isolated afterwards, classified into four groups in five years (strains 1–5: samples collected from 1900–2000; strains 6–22: 2001–2005; strains 23–49: 2006–2010; strains 50–75: 2011–2016). Strain 1: Kresse; Strain 2: Germany IDT; Strain 3: NADL2; Strain 4: Challenge; Strain 5: China 1989; Accession numbers: U44978.1, AY684872.1, NC_001718.1, AY684866.1, HM989009.1.

Similarity	Strains 6–22	Strains 23–49	Strains 50–75
Nucleotide strains (1–5)	98.9853%	99.0555%	99.1969%
Amino acid strains (1–5)	98.0223%	98.2933%	98.5731%

## Supplementary Materials

The following are available online at www.mdpi.com/1999-4915/9/8/196/s1, Table S1: Result of retrospective detection of PPV in nine provinces of South Korea from March 2013 to March 2017; Table S2: List of NS1, VP1, and VP2 sequences used in this study. The strain isolated in South Korea 2016 was named T142_South Korea in this study; Table S3: Additional data about similarity values of nucleotides and amino acids between strain 1–5 and other 70 strains (back data for table 4) (Strain 1–5: sample collected 1900–2000, Strain 6–22: 2001–2005, Strain 23–49: 2006–2010, Strain 50–75: 2011–2016) (Strain 1: Kresse, Strain 2: Germany IDT, Strain 3: NADL2, Strain 4: Challenge, Strain 5: China 1989; Accession numbers: U44978.1, AY684872.1, NC_001718.1, AY684866.1, HM989009.1); Table S4-1: Detailed information about 28 highly variant regions of amino acid substitutions made in *VP2* gene of strain Kresse and 74 other strains; Table S4-2: Detailed information about 28 highly variant regions of amino acid substitutions made in *VP2* gene of strain NADL-2 and 74 other strains.
