# Supplementary material for: Perspectives on the Evolution of Porcine Parvovirus
Source: Viruses. 2017 Jul 26;9(8):196. doi: 10.3390/v9080196 (PMC5580453; doi:10.3390/v9080196)
Supplement: Supplementary file 1 [file viruses-09-00196-s001.pdf]

Supplementary Table 1. Result of retrospective detection of PPV in nine provinces of South Korea from March 2013 to March 2017.

	Sampling years			
Sampling sites	2013	2014	2015	2016
Gyeonggi	51	10	28	79
Gangwon	25	4	14	30
Chungnam	29	5	20	28
Chungbuk	17	7	13	27
Jeonbuk	22	5	9	19
Jeonnam	19	4	17	33
Gyeongbuk	23	4	15	30
Gyeongnam	28	6	16	25
Jeju	11	0	10	18
Total	225	45	142	289

Supplementary Table 2. List of NS1, VP1, and VP2 sequences used in this study. The strain isolated in South Korea 2016 was named T142_South Korea in this study.

	Gene	Country	Year	Accession no.
1	*NS1, VP1, VP2*	South Korea	2016	KY994646
2	*NS1, VP1, VP2*	South Korea	2003	AY390557.1
3	*NS1, VP1, VP2*	China	2004	AY583318.1
4	*NS1, VP1, VP2*	China	2006	EU790641.1
5	*NS1, VP1, VP2*	China	2013	KX242359.1
6	*NS1, VP1, VP2*	China	2015	KX233726.1
7	*NS1, VP1, VP2*	USA	1976	NC_001718.1
8	*NS1, VP1, VP2*	China	1989	HM989009.1
9	*NS1, VP1, VP2*	China	2007	KJ201928.1
10	*NS1, VP1, VP2*	China	2009	KJ201927.1
11	*NS1, VP1, VP2*	China	2013	KF742500.2
12	*NS1, VP1, VP2*	China	2008	JX992846.1
13	*NS1, VP1, VP2*	China	2010	JN968975.1
14	*NS1, VP1, VP2*	China	2011	JN860197.1
15	*NS1, VP1, VP2*	China	2010	JN872448.1
16	*NS1, VP1, VP2*	Austria	2009	JN400519.1
17	*NS1, VP1, VP2*	Germany	2009	JN400518.1
18	*NS1, VP1, VP2*	Germany	2009	JN400517.1
19	*NS1, VP1, VP2*	Germany	2009	JN400516.1
20	*NS1, VP1, VP2*	USA	2006	GQ884047.1
21	*NS1, VP1, VP2*	USA	2006	GQ884046.1

22	*NS1, VP1, VP2*	USA	2006	GQ884045.1
23	*NS1, VP1, VP2*	Europe	2006	GQ884044.1
24	*NS1, VP1, VP2*	Europe	2006	GQ884043.1
25	*NS1, VP1, VP2*	Europe	2006	GQ884042.1
26	*NS1, VP1, VP2*	Europe	2006	GQ884041.1
27	*NS1, VP1, VP2*	Europe	2005	GQ884040.1
28	*NS1, VP1, VP2*	Europe	2005	GQ884039.1
29	*NS1, VP1, VP2*	Europe	2005	GQ884038.1
30	*NS1, VP1, VP2*	Europe	2005	GQ884037.1
31	*NS1, VP1, VP2*	Europe	2005	GQ884036.1
32	*NS1, VP1, VP2*	Europe	2006	GQ884035.1
33	*NS1, VP1, VP2*	USA	1985	U44978.1
34	*NS1, VP1, VP2*	China	2004	EU790642.1
35	*NS1, VP1, VP2*	China	2006	DQ675456.1
36	*NS1, VP1, VP2*	Germany	2002	AY684869.1
37	*NS1, VP1, VP2*	China	2008	FJ822038.1
38	*NS1, VP1, VP2*	Germany	1964	AY684872.1
39	*NS1, VP1, VP2*	United Kingdom	1986	AY684866.1
40	*NS1, VP1, VP2*	China	2013	KM268633.1
41	*NS1, VP1, VP2*	China	2009	HM627653.1
42	*NS1, VP1, VP2*	China	2010	HM627652.1
43	*NS1*	China	2003	AY502114.1
44	*NS1*	China	2013	KF429255.1
45	*NS1*	China	2013	KF429254.1
46	*NS1*	China	2012	KF429253.1
47	*NS1*	China	2012	KF429252.1

48	*NS1*	China	2011	JQ085425.1
49	*NS1*	Denmark	2011	JX568153.1
50	*NS1*	China	2010	JQ710893.1
51	*NS1*	China	2011	JQ710892.1
52	*NS1*	China	2010	JQ710891.1
53	*NS1*	China	2011	JQ710890.1
54	*NS1*	China	2011	JQ710889.1
55	*NS1*	China	2011	JQ710888.1
56	*NS1*	China	2010	JQ710887.1
57	*NS1*	China	2010	JQ710886.1
58	*NS1*	Germany	1999	JN400525.1
59	*NS1*	Switzerland	2009	JN400524.1
60	*NS1*	Switzerland	2009	JN400523.1
61	*NS1*	Germany	2002	JN400522.1
62	*NS1*	Germany	2001	JN400521.1
63	*NS1*	China	2006	DQ499631.2
64	*NS1*	China	2008	FJ853420.1
65	*NS1*	China	2008	EU707335.1
66	*NS1*	China	2012	JX871883.1
67	*NS1*	China	2004	AY789534.1
68	*NS1*	China	2004	AY789533.1
69	*NS1*	China	2004	AY789532.1
70	*NS1*	China	2004	AY686601.1
71	*NS1*	China	2010	HM355808.1
72	*VP1, VP2*	Romania	2011	JQ249926.1
73	*VP1, VP2*	Romania	2011	JQ249927.1
74	*VP1, VP2*	Romania	2011	JQ249925.1

75	*VP1, VP2*	Romania	2011	JQ249924.1
76	*VP1, VP2*	Romania	2011	JQ249923.1
77	*VP1, VP2*	Romania	2011	JQ249922.1
78	*VP1, VP2*	Romania	2011	JQ249921.1
79	*VP1, VP2*	Romania	2011	JQ249920.1
80	*VP1, VP2*	Romania	2011	JQ249919.1
81	*VP1, VP2*	Romania	2011	JQ249918.1
82	*VP1, VP2*	Romania	2011	JQ249917.1
83	*VP1, VP2*	Romania	2011	JQ249916.1
84	*VP1, VP2*	Romania	2011	JQ249915.1
85	*VP1, VP2*	Romania	2011	JQ249914.1
86	*VP1, VP2*	Romania	2011	JQ249913.1
87	*VP1, VP2*	Germany	2001	AY684871.1
88	*VP1, VP2*	Switzerland	2009	JN400520.1
89	*VP1, VP2*	Germany	2002	AY684870.1
90	*VP1, VP2*	Germany	2001	AY684868.1
91	*VP1, VP2*	Germany	2002	AY684867.1
92	*VP1, VP2*	Germany	2001	AY684865.1
93	*VP1, VP2*	Germany	2002	AY684864.1
94	*VP1, VP2*	Germany	2010	KC296746.1
95	*VP1, VP2*	Germany	2010	KC296745.1
96	*VP1, VP2*	Germany	2010	KC296744.1
97	*VP1, VP2*	Germany	2010	KC296743.1
98	*VP2*	China	2010	JQ686669.1
99	*VP2*	China	2004	AY686602.1
100	*VP2*	China	2006	DQ464345.1
101	*VP2*	China	2008	FJ853421.1

102	*VP2*	China	2003	AY502115.1
103	*VP2*	China	2003	AY459350.1
104	*VP2*	China	2010	HM355807.1

Supplementary Table 3. Additional data about similarity values of nucleotides and amino acids between strain 1–5 and other 70 strains (back data for table 4) (Strain 1–5: sample collected 1900–2000, Strain 6–22: 2001–2005, Strain 23–49: 2006–2010, Strain 50–75: 2011–2016). (Strain 1: Kresse, Strain 2: Germany IDT, Strain 3: NADL2, Strain 4: Challenge, Strain 5: China 1989; Accession numbers: U44978.1, AY684872.1, NC_001718.1, AY684866.1, HM989009.1)

Nucleotide

	**6**	**7**	**8**	**9**	**10**	**11**	**12**	**13**	**14**	**15**	**16**	**17**	**18**	**19**	**20**	**21**	**22**
1	98.4	98.3	99.3	99.7	99.2	99.4	98.4	98.4	97.7	97.8	98.3	98.3	98.4	98.3	98.3	98.3	99.0
2	99.3	99.1	98.8	98.7	98.6	98.9	99.0	99.3	98.5	98.6	99.1	99.3	99.2	99.3	99.3	99.3	98.8
3	99.5	99.4	99.0	98.9	99.0	99.1	99.2	99.6	98.6	98.7	99.5	99.5	99.6	99.5	99.5	99.5	99.0
4	99.4	99.2	98.9	98.7	98.8	98.9	99.0	99.4	98.7	98.7	99.3	99.3	99.4	99.3	99.3	99.3	98.9
5	99.4	99.3	98.7	98.6	98.7	98.8	99.1	99.3	98.5	98.6	99.2	99.3	99.3	99.3	99.3	99.3	98.7

	**23**	**24**	**25**	**26**	**27**	**28**	**29**	**30**	**31**	**32**	**33**	**34**	**35**	**36**	**37**	**38**	**39**	**40**	**41**	**42**	**43**	**44**	**45**	**46**	**47**	**48**	**49**
1	98.6	98.5	98.7	98.5	98.4	97.9	98.5	98.4	98.3	98.2	98.1	98.5	97.7	98.3	98.1	98.4	98.9	98.2	98.3	98.4	98.4	98.3	98.8	98.3	99.2	98.4	98.9
2	99.4	99.3	99.4	99.6	99.3	98.9	99.3	99.3	99.3	99.0	98.9	99.6	98.5	99.1	98.9	99.1	98.6	99.1	99.1	99.3	99.3	99.4	99.4	99.2	98.7	99.3	98.7
3	99.7	99.7	99.8	99.5	99.6	99.1	99.6	99.6	99.5	98.9	98.9	99.5	98.6	99.5	98.9	99.5	98.7	99.4	99.3	99.5	99.5	99.4	99.7	99.4	99.0	99.5	98.8
4	99.5	99.5	99.6	99.5	99.4	98.9	99.4	99.4	99.3	98.9	98.8	99.5	98.7	99.3	98.8	99.3	98.5	99.2	99.1	99.4	99.4	99.3	99.5	99.3	98.8	99.4	98.6
5	99.4	99.3	99.4	99.9	99.3	98.9	99.3	99.3	99.3	99.0	98.9	99.7	98.5	99.2	98.9	99.2	98.4	99.2	99.1	99.4	99.4	99.5	99.5	99.3	98.6	99.4	98.6

	**50**	**51**	**52**	**53**	**54**	**55**	**56**	**57**	**58**	**59**	**60**	**61**	**62**	**63**	**64**	**65**	**66**	**67**	**68**	**69**	**70**	**71**	**72**	**73**	**74**	**75**

1	98.3	98.6	98.5	99.0	98.4	98.6	98.7	98.4	98.6	98.6	98.3	98.7	98.3	98.6	98.7	98.7	98.4	98.4	98.4	98.4	98.3	98.4	97.8	98.3	98.7	98.7
2	99.3	99.3	99.3	98.7	99.0	99.4	98.9	99.3	99.4	99.4	99.3	99.3	99.3	99.4	99.3	99.3	99.5	99.5	99.4	99.5	99.5	99.6	98.4	99.5	99.3	99.3
3	99.5	99.6	99.5	99.0	99.2	99.8	99.3	99.5	99.8	99.7	99.5	99.7	99.5	99.8	99.6	99.7	99.4	99.4	99.4	99.5	99.2	99.5	98.9	99.4	99.7	99.7
4	99.3	99.4	99.4	98.8	99.0	99.6	99.1	99.4	99.6	99.5	99.3	99.5	99.3	99.6	99.4	99.5	99.4	99.4	99.3	99.4	99.3	99.5	98.7	99.4	99.5	99.5
5	99.3	99.3	99.2	98.6	99.0	99.5	98.9	99.4	99.5	99.4	99.3	99.4	99.3	99.5	99.3	99.4	99.8	99.9	99.5	99.6	99.7	99.7	98.5	99.7	99.4	99.4

Amino acid

	6	7	8	9	10	11	12	13	14	15	16	17	18	19	20	21	22
1	97.4	97.2	98.4	99.0	98.1	98.8	97.4	97.1	95.0	95.2	97.6	96.9	97.8	97.2	97.2	97.2	98.1
2	98.6	98.4	98.4	98.3	97.6	98.3	97.8	98.3	96.0	96.2	98.8	98.1	98.8	98.4	98.4	98.4	98.1
3	99.5	99.3	98.8	98.3	98.4	98.4	98.3	99.1	96.4	96.5	99.7	99.0	99.8	99.3	99.3	99.3	98.4
4	98.8	98.6	98.1	97.6	97.8	97.8	97.6	98.4	96.2	96.4	99.0	98.3	99.1	98.6	98.6	98.6	97.8
5	98.6	98.4	97.8	97.6	97.6	97.6	97.9	98.3	96.0	96.2	98.8	98.1	98.8	98.4	98.4	98.4	97.4

	23	24	25	26	27	28	29	30	31	32	33	34	35	36	37	38	39	40	41	42	43	44	45	46	47	48	49
1	97.6	97.8	97.9	97.2	97.8	96.7	97.6	97.8	97.2	97.1	96.9	97.2	95.0	96.7	96.9	97.4	98.3	97.2	97.2	97.2	97.2	97.2	98.1	97.2	98.3	97.2	98.4
2	98.8	98.8	99.0	99.0	99.0	97.9	98.8	99.0	98.4	98.1	97.8	99.0	96.0	97.9	97.8	98.6	98.3	98.4	98.4	98.4	98.4	98.8	99.1	98.4	98.3	98.4	98.4
3	99.7	99.8	100	99.0	99.8	99.8	99.7	99.8	99.3	97.8	97.6	99.0	96.4	98.8	97.6	99.5	98.6	99.3	99.3	99.3	99.3	98.8	99.8	99.3	98.6	99.3	98.8
4	99.0	99.1	99.3	98.6	99.1	98.1	99.0	99.1	98.6	97.4	97.2	98.6	96.2	98.1	97.2	98.8	97.9	98.6	98.6	98.6	98.6	98.4	99.1	98.6	97.9	98.6	98.1
5	98.8	98.8	99.0	99.7	99.0	97.9	98.8	99.0	98.4	97.6	97.4	99.0	96.0	97.0	97.4	98.6	97.6	98.4	98.4	98.4	98.4	98.8	99.1	98.4	97.6	98.4	97.8

	**50**	**51**	**52**	**53**	**54**	**55**	**56**	**57**	**58**	**59**	**60**	**61**	**62**	**63**	**64**	**65**	**66**	**67**	**68**	**69**	**70**	**71**	**72**	**73**	**74**	**75**
1	97.2	97.9	98.1	98.1	97.8	97.8	97.8	97.2	97.8	97.6	97.2	97.8	97.2	97.6	97.8	97.9	97.2	97.4	97.2	97.2	97.2	97.2	95.7	96.9	97.8	97.9
2	98.4	99.0	99.1	98.1	99.0	99.0	98.8	98.4	99.0	98.8	98.4	99.0	98.4	98.8	99.0	99.0	98.6	98.8	98.6	99.0	99.0	99.0	96.9	98.6	98.8	99.0
3	99.3	99.7	99.8	98.4	99.8	99.8	99.5	99.3	99.8	99.7	99.3	99.8	99.3	99.7	99.8	100	98.6	98.6	98.4	99.0	98.4	99.0	97.8	98.6	99.8	100
4	98.6	99.0	99.1	97.8	99.1	99.1	98.8	98.6	99.1	99.0	98.6	99.1	98.6	99.0	99.1	99.3	98.3	98.3	98.1	98.6	98.1	98.6	97.1	98.3	99.1	99.3
5	98.4	98.6	98.8	97.4	99.0	99.0	98.4	98.4	99.0	98.8	98.4	99.0	98.4	98.8	99.0	99.0	99.3	99.7	98.4	99.0	99.1	99.0	96.9	99.0	98.8	99.0

Supplementary Table 4-1. Detailed information about 28 highly variant regions of amino acid substitutions made in *VP2* gene of strain Kresse and 74 other strains.

AA	1	2	3	4	5	6	7	8	9	10	11	12	13	14	15	16	17	18	19	20	21	22	23	24	25
20	A						A	A	A	A											A				
45		T																							
82	K						K	K	K	K											K				
93	E							E	E	E															
101													G	G											
144				A																					A
215		I	I	I									V	V											I
226																					E				
228					E	E										E		E	E	E					
233							T	T	T	T	T	T	A	A							T				
238			P										P	P											
304	T						T	T	T	T															
320	T	T					T	T		T	M		T	T							T				
366							N														N				
378											D														
383	H			H							H		H	H											
391												V	V	V											
407	N	N					N	N		N											N				
414						S			S							S		S	S	S					
419					Q	Q			Q							Q		Q	Q	Q	Q				
436		S		S	T	T			T	T		A	A	A	A	T		T	T	T		T			S
439													Y	Y											

521												H	H	H											
550													P	P											
555	K	K		K				K			K														K
564	M							M			M														
565													R	R											
570													H	H											

AA	26	27	28	29	30	31	32	33	34	35	36	37	38	39	40	41	42	43	44	45	46	47	48	49	50
20													A								A		A		
45						G												T							
82													K								K		K		
93																									
101									G																
144								A																	
215						I	I	I	V		I							I							
226													E								E				
228		E			E									E	E	E	E			E		E		E	
233									A			T	T								T			T	
238									P																
304													T					S			T		T		
320						T	T	T	T		T		T								T		T		T
366													N								N		N		
378								D										D							
383						H	H		H		H							H							
391						V	V		V	V	V														
407						N	N				N		N								N		N		
414		S			S							S		S	S	S	S			S		S		S	

419		Q			Q									Q	Q	Q	Q			Q		Q		Q	
436	A	T	A	T	T	A	A	S	A	T	A	A		T	T	T	T	S		T		T		T	
439									Y																
521									H	H															
550									P	P															
555																			K						
564						M	M				M														
565		E						R	R									R							
570						H	H		H	H	H														

AA	51	52	53	54	55	56	57	58	59	60	61	62	63	64	65	66	67	68	69	70	71	72	73	74
20		A																						
45																	T	T	T	T		T		
82		K																						
93																								
101																								
144															A	A						A		
215															I	I	I	I	I	I		I		
226																								
228		E				E		E	E		E													
233		T			T							T												
238																								
304		T																						
320	T	T															M							
366		N			N																			
378															D		D	D	X	D		D		

383															H	H	H	H	H	H		H		
391																					V			
407		N			N											M			M					
414						S			S		S													
419						Q			Q		Q													
436			T	T		T	T	A	T	A	T	A	A		S	S	S	S	S	S	H	S		
439																								
521																								
550																								
555															K	K			K					
564																	M							
565																	R	R		R		R		
570																								

Supplementary Table 4-2. Detailed information about 28 highly variant regions of amino acid substitutions made in *VP2* gene of strain NADL-2 and 74 other strains.

AA	1	2	3	4	5	6	7	8	9	10	11	12	13	14	15	16	17	18	19	20	21	22	23	24	25
20	A						A	A	A	A											A				
45	S	S	S	S	S	S	S	S	S	S	S	S	S	S	S	S	S	S	S	S	S	S	S	S	S
82	K						K	K	K	K											K				
93	E							E	E	E															
101													G	G											
144				A																					A
215	T	T			T	T	T	T	T	T	T	T	V	V	T	T	T	T	T	T	T	T	T	T	
226																					E				
228					E	E										E		E	E	E					
233							T	T	T	T	T	T	A	A							T				
238			P										P	P											
304	T						T	T	T	T											T				
320		I	I	I	I	I			I		M	I		I	I	I	I	I	I			I	I	I	I
366							N														N				
378											D														
383	H			H							H		H	H											
391												V	V	V											
407		K	K	K	K	K			K		K	K	K	K	K	K	K	K	K	K		K	K	K	K
414						S			S							S		S	S	S					
419					Q	Q			Q							Q		Q	Q	Q	Q				
436	P	P	P		T	T	P	P	T	T	P	A	A	A	A	T	P	T	T	T	P	T	P	P	
439													Y	Y											

521												H	H	H											
550													P	P											
555		N	N		N	N	N		N	N		N	N	N	N	N	N	N	N	N	N	N	N	N	
564	M							M			M														
565													R	R											
570													H	H											

AA	26	27	28	29	30	31	32	33	34	35	36	37	38	39	40	41	42	43	44	45	46	47	48	49	50
20													A								A		A		
45	S	S	S	S	S	G	S	S	S	S	S	S	S	S	S	S	S		S	S	S	S	S	S	S
82													K								K		K		
93																									
101									G																
144								A																	
215	T	T	T	T	T				V	T		T	T	T	T	T	T		T	T	T	T	T	T	T
226													E								E				
228		E			E									E	E	E	E			E		E		E	
233									A			T	T								T		T		
238									P																
304													T					S			T		T		
320	I	I	I	I	I					I		I		I	I	I	I	I	I	I		I		I	
366													N								N		N		
378								D										D							
383						H	H		H		H							H							
391						V	V		V	V	V														
407	K	K	K	K	K			K	K	K		K		K	K	K	K	K	K	K		K		K	K
414		S			S							S		S	S	S	S			S		S		S	

419		Q			Q									Q	Q	Q	Q			Q		Q		Q	
436	A	T	A	T	T	A	A		A	T	A	A	P	T	T	T	T		P	T	P	T	P	T	P
439									Y																
521									H	H															
550									P	P															
555	N	N	N	N	N	N	N	N	N	N	N	N	N	N	N	N	N	N		N	N	N	N	N	N
564						M	M				M														
565		E						R	R									R							
570						H	H		H	H	H														

AA	51	52	53	54	55	56	57	58	59	60	61	62	63	64	65	66	67	68	69	70	71	72	73	74
20		A																						
45	S	S	S	S	S	S	S	S	S	S	S	S	S	S	S	S					S		S	S
82		K																						
93																								
101																								
144															A	A						A		
215	T	T	T	T	T	T	T	T	T	T	T	T	T	T							T		T	T
226																								
228		E				E		E	E		E													
233		T			T							T												
238																								
304		T																						
320			I	I	I	I	I	I	I	I	I	I	I	I	I	I	M	I	I	I	I	I	I	I
366		N			N																			

378															D		D	D	X	D		D		
383															H	H	H	H	H	H		H		
391																					V			
407	K		K	K		K	K	K	K	K	K	K	K	K	K	M	K	K	M	K	K	K	K	K
414						S			S		S													
419						Q			Q		Q													
436	P	P	T	T	P	T	T	A	T	A	T	A	A	P							H		P	P
439																								
521																								
550																								
555	N	N	N	N	N	N	N	N	N	N	N	N	N	N			N	N		N	N	N	N	N
564																	M							
565																	R	R		R		R		
570																								
